# Notes on the genus *Pedionis* Hamilton (Hemiptera, Cicadellidae, Macropsinae), and with description of two new species from China

**DOI:** 10.3897/zookeys.96.1495

**Published:** 2011-05-10

**Authors:** Hu Li, Renhuai Dai, Zizhong Li

**Affiliations:** Institute of Entomology, Guizhou University; The Provincial Key Laboratory for Agricultural Pest Management of Mountainous Region, Guiyang, Guizhou, P.R. China, 550025

**Keywords:** Hemiptera, Macropsinae, *Pedionis*, new species, taxonomy, distribution, China

## Abstract

Two new species *Pedionis (Pedionis) nankunshanensis* Li, Dai & Li **sp. n.** and *Pedionis (Pedionis) tabulatus* Li, Dai & Li **sp. n.** from China are described and illustrated. A key is given to separate all species of this genus (except *Pedionis (Pedionis) oeroe* and *Pedionis (Pedionis) thyia*).

## Introduction

The leafhopper genus *Pedionis* belongs to the tribe Macropsini, subfamily Macropsinae (Hemiptera: Cicadellidae) and was established by Hamilton (1980) with *Pediopsis garuda* Distant, 1916 as its type species. Hamilton separated two subgenera *Pedionis* and *Thyia* by anteapical cells and proposed six new combinations (one in subgenus *Thyia*) and described three new species. Later, 12 new species (Viraktamath 1981, 1996; Kuoh 1987; Huang and Virakatamath 1993; Liu and Zhang 2003; Zhang and Viraktamath 2010) were described and illustrated. Currently 20 species of the subgenus *Pedionis* and 1 species of the subgenus *Thyia* have been recorded, and 8 of the subgenus *Pedionis* from China.

Species of *Pedionis* are mainly distributed in the Oriental region, and almost half of them (all belong to subgenus *Pedionis*) are found in southern of China (Oriental region) and most *Pedionis* species are associated with shrubs and trees.

In this paper, two new Chinese species of the genus from Guizhou and Guangdong Province are described and illustrated. 23 species of the genus and a key to species from the world is provided (except *Pedionis (Pedionis) oeroe* and *Pedionis (Pedionis) thyia*). The type specimens of the new species are deposited in the Institute of Entomology, Guizhou University, Guiyang, China (GUGC).

## Taxonomy

### Genus Pedionis

Hamilton

Pedionis Hamilton 1980: 891.

#### Type species:

 *Pediopsis garuda* Distant, 1916.

#### Diagnosis.

Following Hamilton (1980).

#### Subgenus Pedionis (Pedionis)

Hamilton

Pedionis (Pedionis) Hamilton 1980: 892.

##### Type species:

 *Pediopsis garuda* Distant, 1916: 239.

##### Diagnosis.

Following Hamilton (1980).

##### Distribution.

Oriental region, Palaearctic region, Northern Australia.

#### Subgenus Pedionis (Thyia)

Hamilton

Pedionis (Thyia) Hamilton 1980: 894.

##### Type species:

 *Macropsis thyia* Kirkaldy, 1907: 36.

##### Diagnosis.

Following Hamilton (1980).

##### Distribution.

Northern Australia.

#### Key to male species of genus Pedionis (except P. (Pedionis) oeroe and P. (Pedionis) thyia)

**Notes:** Thespecies *Pedionis (Pedionis) oeroe* should belong to the subgenus *Pedionis* by tegmina with only 2 subapical cells and veins dark fuscous multiannulate with whitish according to the original description, but no male genitalia manuscript (Kirkaldy 1907), the species *Pedionis (Pedionis) thyia* is distinguished from others by amount of anteapical cells (non-genitalic characters), therefore, the key don’t encompass these two species.

**Table:** 

1	Aedeagal shaft without any processes (Figs 9–10, 15–16, 33–34, 37)	2
–	Aedeagal shaft with 1–2 processes (Figs 1–8, 11–14, 17–32, 35–36, 47–48, 55–56)	5
2	Aedeagus broader basally and tapering apically (Figs 9–10, 33–34)	3
–	Aedeagus less broader basally and about the end (Figs 15–16, 37)	4
3	Gonopore opening on the apex of adeagal shaft (Figs 9–10)	*Pedionis (Pedionis) curvata*
–	Gonopore opening on the subapical of adeagal shaft (Figs 33–34)	*Pedionis (Pedionis) venosa*
4	Aedeagal shaft strongly sinuated (Fig. 37)	*Pedionis (Pedionis) minuta*
–	Aedeagal shaft less sinuated and with protuberance in middle-dorsal aspect (Figs 15–16)	*Pedionis (Pedionis) koghiensis*
5	Aedeagal shaft with one pair of processes (Figs 1–8, 17–22, 25–26, 29–30)	6
–	Aedeagal shaft with two pairs of processes (Figs 11–14, 23–24, 27–28, 31–32, 35–36, 47–48, 55–56)	14
6	This one pair of processes produced on the apex of aedeagal shaft (Figs 3–4, 17–22, 25–26, 29–30)	7
–	This one pair of processes produced on the subapical of aedeagal shaft (Figs 1–2, 5–8)	12
7	The processes situated on the ventral margin of apical aedeagal shaft (Figs 3–4, 17–22, 25–26)	8
–	The processes situated on the dorsal margin of apical aedeagal shaft (Figs 29–30)	*Pedionis (Pedionis) stigma*
8	The apex of aedeagal as arrow-like (Figs 17–20)	9
–	The apex of aedeagal as curved-like or serrated (Figs 3–4, 21–22, 25–26)	10
9	Aedeagal shaft with a bulbous (Figs 19–20)	*Pedionis (Pedionis) mecota*
–	Aedeagal shaft without any bulbous, tapering apically (Figs 17–18)	*Pedionis (Pedionis) lii*
10	Apex of aedeagal shaft as curved-like (Figs 21–22, 25–26)	11
–	Apex of aedeagal shaft as serrated-like (Figs 3–4)	*Pedionis (Pedionis) cherraensis*
11	Aedeagal shaft with a bulge nearly base, the lateral aspect of aedeagus strongly sinuated (Figs 25–26)	*Pedionis (Pedionis) serrate*
–	Aedeagal shaft with a bulge nearly middle, the lateral aspect of aedeagus less sinuated (Figs 21–22)	*Pedionis (Pedionis) palniensis*
12	The pair processes wide and as serrated (Figs 5–6)	*Pedionis (Pedionis) clypellata*
–	The pair processes narrow and as lamella (Figs 1–2, 7–8)	13
13	Aedeagal shaft with a constriction in middle, the lateral aspect of aedeagus strongly sinuated (Figs 1–2)	*Pedionis (Pedionis) astrala*
–	Aedeagal shaft without any constriction in any position, the lateral aspect of aedeagus less sinuated (Figs 7–8)	*Pedionis (Pedionis) contrasta*
14	Two pairs of processes without connection, separated (Figs 23–24, 27–28, 31–32, 35–36, 55–56)	15
–	Two pairs of processes with a membranous connection (Figs 11–14, 47–48)	19
15	The first pair of processes produced on the dorsal margin of apical aedeagal shaft, the second wide (Figs 55–56)	*Pedionis (Pedionis) tabulatus* Li, Dai & Li sp. n.
–	The first pair of processes produced on the ventral margin of apical aedeagal shaft, the second narrow (Figs 23–24, 27–28, 31–32, 35–36)	16
16	The second pair of processes closely to the first (Figs 35–36)	*Pedionis (Pedionis) yunnana*
–	The second pair of processes away from the first (Figs 23–24, 27–28, 31–32)	17
17	Dorsal aspect of aedeagal shaft with protuberance in middle (Figs 27–28, 31–32)	18
–	Dorsal aspect of aedeagal shaft without protuberance (Figs 23–24)	*Pedionis (Pedionis) rufoscutallata*
18	Aedeagal shaft with a bulge nearly middle, the second pair of processes towards dorsal aspect (Figs 31–32)	*Pedionis (Pedionis) sumatrana*
–	Aedeagal shaft without a bulge nearly middle, the second pair of processes towards ventral aspect (Figs 27–28)	*Pedionis (Pedionis) spinata*
19	The first pair of processes produced on the dorsal margin of aedeagal shaft as serrated, the second have reflexed ventral aspect view (Figs 47–48)	*Pedionis (Pedionis) nankunshanensis* Li, Dai & Li sp. n.
–	The first pair of processes produced on the ventral margin of aedeagal shaft, the second have no reflexed ventral aspect view (Figs 11–14)	20
20	The second pair of processes wide basally, aedeagal shaft strongly sinuated (Figs 13–14)	*Pedionis (Pedionis) kagoshimensis*
–	The second pair of processes slender, aedeagal shaft less sinuated (Figs 11–12)	*Pedionis (Pedionis) garuda*

**Figures 1–37.**
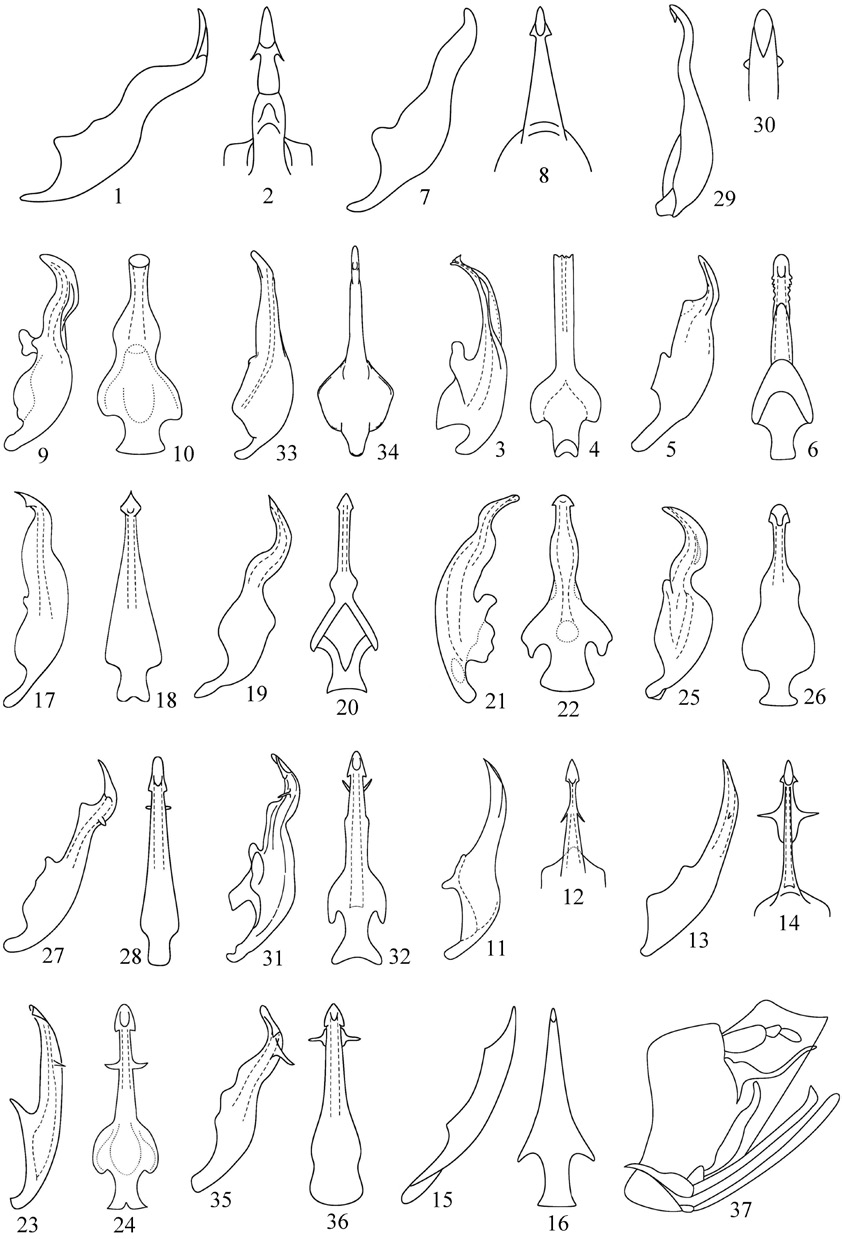
Aedeagus of *Pedionis* species, lateral view and ventral view. **1, 2** *Pedionis astrala* Hamilton **3, 4** *Pedionis cherraensis* Viraktamath **5, 6** *Pedionis clypellata* Huang & Viraktamath **7, 8** *Pedionis contrasta* Hamilton **9, 10** *Pedionis curvata* Viraktamath **11, 12** *Pedionis garuda* (Distant) **13, 14** *Pedionis kagoshimensis* (Matsumura) **15, 16** *Pedionis koghiensis* (Evans) **17, 18** *Pedionis lii* Zhang & Viraktamath **19, 20** *Pedionis mecota* Liu & Zhang **21, 22** *Pedionis palniensis* Viraktamath **23, 24** *Pedionis rufoscutallata* Huang & Viraktamath **25, 26** *Pedionis serrate* Viraktamath **27, 28** *Pedionis spinata* Zhang&Viraktamath **29, 30** *Pedionis stigma* Kouh **31, 32** *Pedionis sumatrana* Viraktamath **33, 34** *Pedionis venosa* Hamilton **35, 36** *Pedionis yunnana* Zhang & Viraktamath **37** *Pedionis minuta* (Evans). (**1–2, 7–8, 13–14** after Hamilton 1980; **3–4, 31–32** after Viraktamath 1996; **5–6, 23–24** after Huang and Viraktamath 1993; **9–12, 21–22, 25–26** after Viraktamath 1981; **15–16** after Evans 1974; **17–18, 27–28; 35–36** after Zhang and Viraktamath 2010; **19–20** after Liu and Zhang 2003; **29–30** after Kuoh 1987; **33–34** after Okudera 2009; **37** after Evans 1971)

**Figures 38–44.**
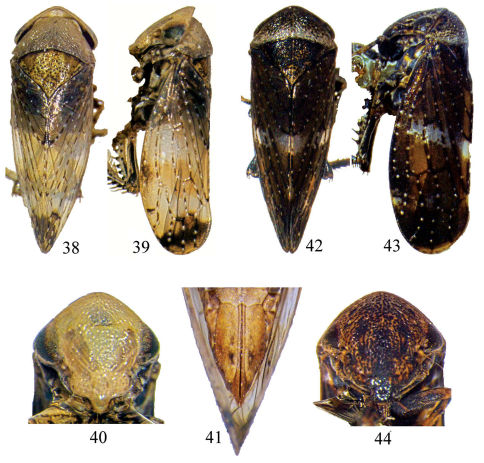
*Pedionis (Pedionis) nankunshanensis* Li, Dai & Li sp. n. **38** Dorsal view, (*♂*) **39** Lateral view, (*♂*) **40** Facial view, (*♂*) **41** Seventh sternite ventral view. **42–44** *Pedionis (Pedionis) tabulatus* Li, Dai & Li sp. n. **42** Dorsal view, (*♂*) **43** Lateral view, (*♂*) **44** Facial view, (*♂*).

#### Pedionis (Pedionis) nankunshanensis

Li, Dai & Li sp. n.

urn:lsid:zoobank.org:act:F6618549-C9A5-4430-8458-43FC0B39DDB0

http://species-id.net/wiki/Pedionis_(Pedionis)_nankunshanensis

Figs 45–52 

##### Description.

Body yellowish-brown (Fig. 38). The vertex inverted “V” shaped, as wide as pronotum (Fig. 38), weakly curved in profile, slightly away from the pronotum (Fig. 39); eyes brown; ocelli located between the eyes, its surrounding yellow, below gray (Fig. 40). The pronotum pale-yellow, anterior margin curved prominent, posterior margin slightly concave. Scutellum triangular, yellowish, scatter dark notches, base-lateral sides gray, post-middle region with one deep notch (Fig. 38). Forewings hyaline, end area chocolate-brown, veins fuscous white spots distinctly (Fig. 39).

**Figures 45–52.**
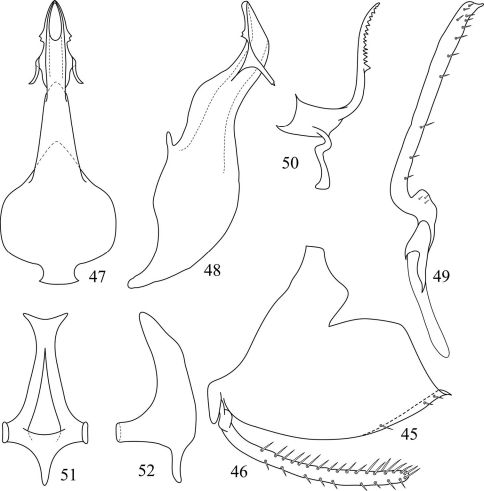
*Pedionis (Pedionis) nankunshanensis*Li, Dai & Li sp. n. **45** Male pygofer side, lateral view **46** Subgenital plate, lateral view **47** Aedeagus, ventral view **48** Aedeagus, lateral view **49** Style, dorsal view **50** Dorsal connective **51** Connective, dorsal view **52** Connective, lateral view.

###### Male genitalia

 Pygofer broad, the apex acute in lateral view and produced several setae on the ventral margin (Fig. 45). Subgenital plate slender with many marginal setae (Fig. 46). Aedeagus broader basally, shaft strongly sinuate in lateral view, apex tapering, and with two pairs of processes, the apical processes located dorsal margin, serrated, the subapical processes located lateral margin, reflexed in ventral aspect view, apex digitation, the processes with a membranous connection (Figs 47–48). Style parallel-margined and angled on the apical third, the apex obliquely truncate, produced a narrow truncate process on dorsal margin (Fig. 49). Dorsal connective complex and sinuate, apex bulbous, produced a long process from caudal margin to dorsad, and mesal-ventral apical margin minutely serrated (Fig. 50). Connective broader basally, a finger-like protrusion in middle, both sides bent to the inside (Figs 51–52).

###### Female.

 Similar to male in coloration and appearance. The seventh sternite 1.5 times the sixth sternite, carved in middle-posterior margin (Fig. 41).

###### Measurement.

Length (including tegmen): *♂*, 3.2–3.5 mm;♀, 3.8–4.0 mm.

##### Type material.

Holotype *♂*, China: Guangdong Prov., Nankunshan, 24 August 2010, collected by Hu Li (GUGC). Paratypes: 1*♂*1♀, same data as holotype; 2♀♀, Guangdong Prov., Nankunshan, 22 August 2010, collected by Junqiang Ni (GUGC).

##### Diagnosis.

This species is similar to *Pedionis (Pedionis) yunnana* Zhang & Viraktamath, 2010 but can be distinguished from the latter by having the apical processes on aedeagal shaft serrated; the subapical processes reflexed ventral aspect view, apex digitations.

##### Etymology.

The new species name refers to the type locality.

#### Pedionis (Pedionis) tabulatus

Li, Dai & Li sp. n.

urn:lsid:zoobank.org:act:F3EBE21A-C693-4E00-94EA-B5FBE7706B78

http://species-id.net/wiki/Pedionis_(Pedionis)_tabulatus

Figs 53–60 

##### Description.

Body coloration and appearance similar to *Pedionis (Pedionis) lii* Zhang & Viraktamath, 2010 but more dark and pronotum slightly concave, with a white belt on posterior margin (Figs 42–44).

###### Male genitalia.

**Figures 53–60.**
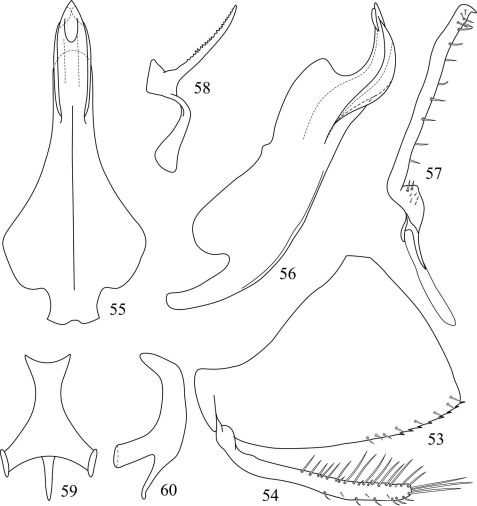
*Pedionis (Pedionis) tabulatus*Li, Dai & Li sp. n. **53** Male pygofer side, lateral view **54** Subgenital plate, lateral view **55** Aedeagus, ventral view **56** Aedeagus, lateral view **57** Style, dorsal view **58** Dorsal connective **59** Connective, dorsal view **60** Connective, lateral view.

Pygofer broad, obliquely truncate, the apex obtuse in lateral view, produced regularly spike-spines and setae on the ventral margin (Fig. 53). Subgenital plate slender with many setae, several especially long in the end (Fig. 54). Aedeagus broader basally, shaft strongly sinuated, angled heavily on apical third and bulge occurred in middle-dorsal in lateral view; apex tapering, and with two pairs of processes, the apical processes small and produced on dorsal margin, the subapical processes located lateral margin, broad as lamella (Figs 55–56). Style (Fig. 57), dorsal connective (Fig. 58) and connective (Figs 59–60) similar to *Pedionis (Pedionis) nankunshanensis* **sp. n.** but differs by mesal-dorsal serration.

###### Female.

Unknown.

###### Measurement.

Length (including tegmen): *♂*, 5.2mm.

##### Type material.

Holotype *♂*, China: Guizhou Prov., Kuankuoshui Nature Reserve, Baishaogou, 7 June 2010, collected by Hu Li (GUGC).

##### Diagnosis.

This species is similar to *Pedionis (Pedionis) yunnana* Zhang & Viraktamath, 2010 but differs markedly from the latter in having the apical processes on aedeagal shaft occurred in dorsal margin; the subapical processes broad, lamella-like; the pygofer with regularly spike-spines and setae on the ventral margin.

##### Etymology.

Th e species name is derived from the Latin words “*tabulatus*”, indicating the subapical processes of adeagal shaft are lamella-like.

## Supplementary Material

XML Treatment for Pedionis
